# Recent Chemical and Chemoenzymatic Strategies to Complex-Type *N*-Glycans

**DOI:** 10.3389/fchem.2022.880128

**Published:** 2022-05-26

**Authors:** Xiaoya Zhao, Yan Huang, Siai Zhou, Jiaming Ao, Hui Cai, Katsunori Tanaka, Yukishige Ito, Akihiro Ishiwata, Feiqing Ding

**Affiliations:** ^1^ School of Pharmaceutical Sciences (Shenzhen), Sun Yat-sen University, Shenzhen, China; ^2^ RIKEN Cluster for Pioneering Research, Saitama, Japan; ^3^ School of Materials and Chemical Technology, Tokyo Institute of Technology, Tokyo, Japan; ^4^ Alexander Butlerov Institute of Chemistry, Kazan Federal University, Kazan, Russian Federation; ^5^ Graduate School of Science, Osaka University, Osaka, Japan

**Keywords:** *N*-glycans, β-mannosylation, α-sialylation, chemical assembly, chemo-enzymatic strategies

## Abstract

Glycosylation is one of the major forms of protein post-translational modification. *N*-glycans attached to proteins by covalent bonds play an indispensable role in intercellular interaction and immune function. In human bodies, most of the cell surface glycoproteins and secreted glycopeptides are modified with complex-type *N*-glycans. Thus, for analytical or medicinal purposes, efficient and universal methods to provide homogeneous complex-type *N*-glycans have been an urgent need. Despite the extremely complicated structures, tremendous progress in the synthesis of *N*-glycans has been achieved. On one hand, chemical strategies are shown to be effective to prepare core oligosaccharides of *N*-glycans by focusing on stereoselective glycosylations such as β-mannosylation and α-sialylation, as well as the methodology of the *N*-glycan assembly. On the other hand, chemoenzymatic strategies have also become increasingly powerful in recent years. This review attempts to highlight the very recent advancements in chemical and chemoenzymatic strategies for eukaryotic complex-type *N*-glycans.

## Graphical Abstract

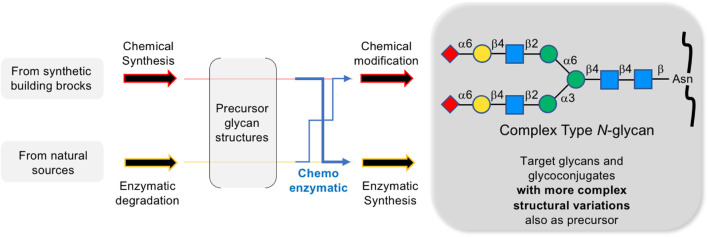

## Introduction

**FIGURE 1:**
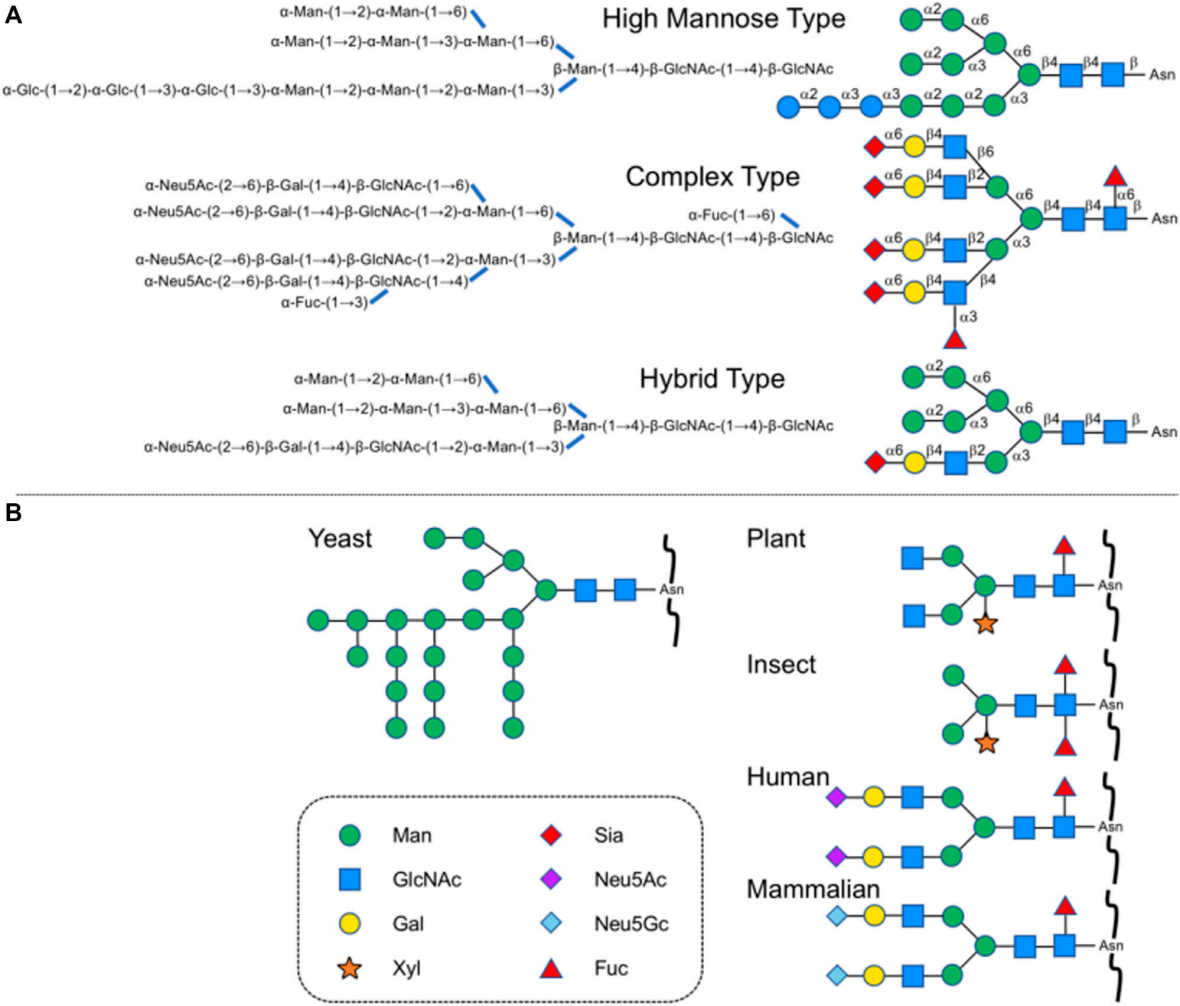
**(A)** Typical *N*-glycan subtypes in yeast and a mammalian cell. **(B)** Typical *N*-glycan structures in other species.

Glycosylation is one of the major post-translational modifications of protein, playing an important role in protein folding, transport, and localization (Varki et al., 2015). It affects protein secretion and stability and participates in cell adhesion and signal transduction. *N*-glycosylation is the most common protein modification, and the attached glycans called *N*-glycans are linked to the asparagine residues in the consensus amino acid sequence (Asn-X-Thr/Ser, X ≠ Pro) at the reducing terminal *N*-acetyl-glucosamine (GlcNAc). According to the differences in non-reducing terminal residues, three subtypes can further be derived, high-mannose type, hybrid type, and complex type. Typical structures are shown in Figure 1A. For medical use, the sialylated complex-type glycans are seen quite often as found in secretory and cell surface glycoproteins. The principal modification can directly improve the *in vivo* stability and physiological activity of glycoproteins, including extending half-life, participating in intermolecular recognition, and enhancing drug targeting compared to the unmodified ones (Reily et al., 2019; Shirakawa et al., 2021b).

In recent years, a variety of glycoproteins have been approved for the treatment and diagnosis of human diseases, such as erythropoietin (EPO), granulocyte colony-stimulating factor (GCSF), β-interferon, certain cancer biomarkers, and monoclonal antibodies for public health purposes as similar biological products (SBPs) with acceptable levels of quality, safety and efficacy (Buettner et al., 2018). Large-scale production has been realized through glycoprotein expression by yeast, plant, insect, and Chinese hamster ovary (CHO) cells, but structures are quite different from those of glycans on human proteins (Figure 1B).

**FIGURE 2:**
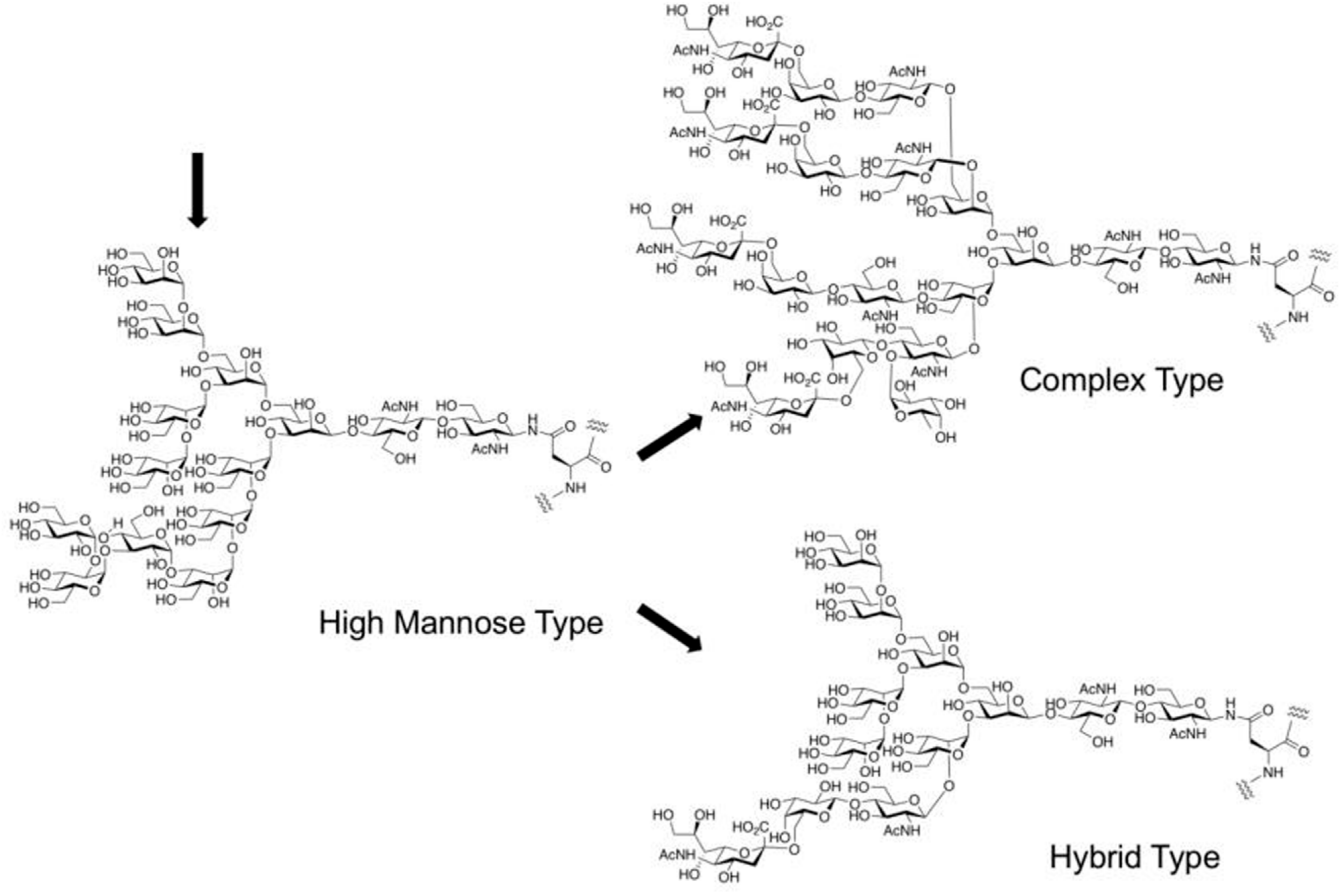
Biosynthesis of *N*-glycan in a cell expression system.

At present, many *N*-linked glycoproteins are produced through biosynthesis of recombinant protein in cell expression systems of insects, plants, yeast, and mammals, in which the required sets of glycosyl transfer systems including enzymes for the preassemble of dolichol pyrophosphate-linked tetradecasaccharide, oligosaccharyltransferase, ER and Golgi glycosidases, UDP glucose glucosyltransferase, and Golgi glycosyltransferases as well as many lectins, have already existed. However, the available proteins obtained by this method are mostly asialo form with lacking other modifications such as fucosylation to *N*-glycan and afford heterogeneous products due to the uncompleted biosynthesis to be a desired *N*-glycan structures (Figure 2) (Vinikangas et al., 2021). Since the heterogeneity of medicinal *N*-glycans affects clinical application, the preparation of humanized and homogeneous glycoforms is important for both research and therapeutic purposes. Meanwhile, isolation from natural resources requires rather complicated protocols with rather difficult purifications and is inadequate for researchers to study diverse structures, especially unnatural forms of *N-*glycans.

**FIGURE 3:**
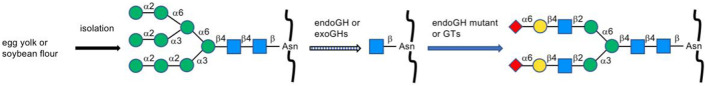
Isolation of *N*-glycans from natural resources.

Isolation of *N*-glycans from natural resources remains one of the most common ways to acquire a large amount of *N*-glycan production (Figure 3). Asn-linked oligosaccharides containing biantennary complex-type *N*-glycans from egg yolk immunoglobulin after hydrazinolysis had been reported (Matsuura et al., 1993). The ammonia-catalyzed release method of ovalbumin and soy protein was reported recently without side reactions and degradation of core *N*-glycan structures (Yang et al., 2020). Sialylglycopeptide (SGP) was also found in hen’s egg yolk and digested by PNGase, one of the most highly specific glycosidases extensively used in enzymatic isolation (Seko et al., 1997). Chemical modification of SGP to various other glycoproteins has been developed by Kajihara’s group, which achieved semi-synthesis of poly-LacNAc containing complex-type biantennary oligosaccharide (Maki et al., 2017) and was recently applied to triantennary erythropoietin (EPO) *N*-glycan (Maki et al., 2020).

## Chemical and Chemoenzymatic Strategies Toward Eukaryotic Complex-Type *N*-Glycans

### Chemical Assembly of Complex-Type *N*-Glycans

As shown in the introduction briefly, the extreme complexity of *N*-glycan structures, especially in terms of the regioselectivity and stereoselectivity control, strongly induces the current situation with no universal methods and strategies for chemical synthesis of *N*-glycan, although there have been widely studied all over the world. One of the issues for the chemical synthesis of *N*-glycan is stereoselective glycosylations including β-D-mannosylation in the core structure of all types of *N*-glycans, and α-sialylation at the non-reducing terminal end of the complex and the hybrid-type *N*-glycans.

The earliest glycosylation reaction dates back to the late 19th century. An example of simple phenolic glycosides synthesis, glycosylation between glycosyl chloride/bromide and nucleophilic potassium phenoxide, was first reported by Michael in 1879 (Michael and Norton, 1879). A great deal of work has been done since then in the development of glycosylation methods and dozens of novel glycosyl donors have been reported in succession, such as halide donors, semi-aldehyde glycosyl donors, trichloroacetimidate donors (TCAI, Schmidt donors), thioglycoside donors, glycal donors, glycosyl phosphate donors, and *o*-acetoxy benzoate glycosyl donors (Koenigs and Knorr, 1901; Danishefsky and Bilodeau, 1996; Garcia and Gin, 2000; Schmidt et al., 1986; Fügedi et al., 1987; Plante et al., 2001). With the emergence of these glycosyl donors, a variety of activation systems have also been developed to optimize the yield and selectivity, which are still playing important roles in the synthesis of various glycans. Among the commonly use donors such as halide donors, Schmidt’s donors, and thioglycosides, it is worth mentioning that, sulfides groups of thioglycosides can act as anomeric protecting groups, and sometimes orthogonal activated leaving groups as well, which give a unique value to thioglycosides, especially in the application to a liquid-phase one-pot synthesis of glycans.

**FIGURE 4:**
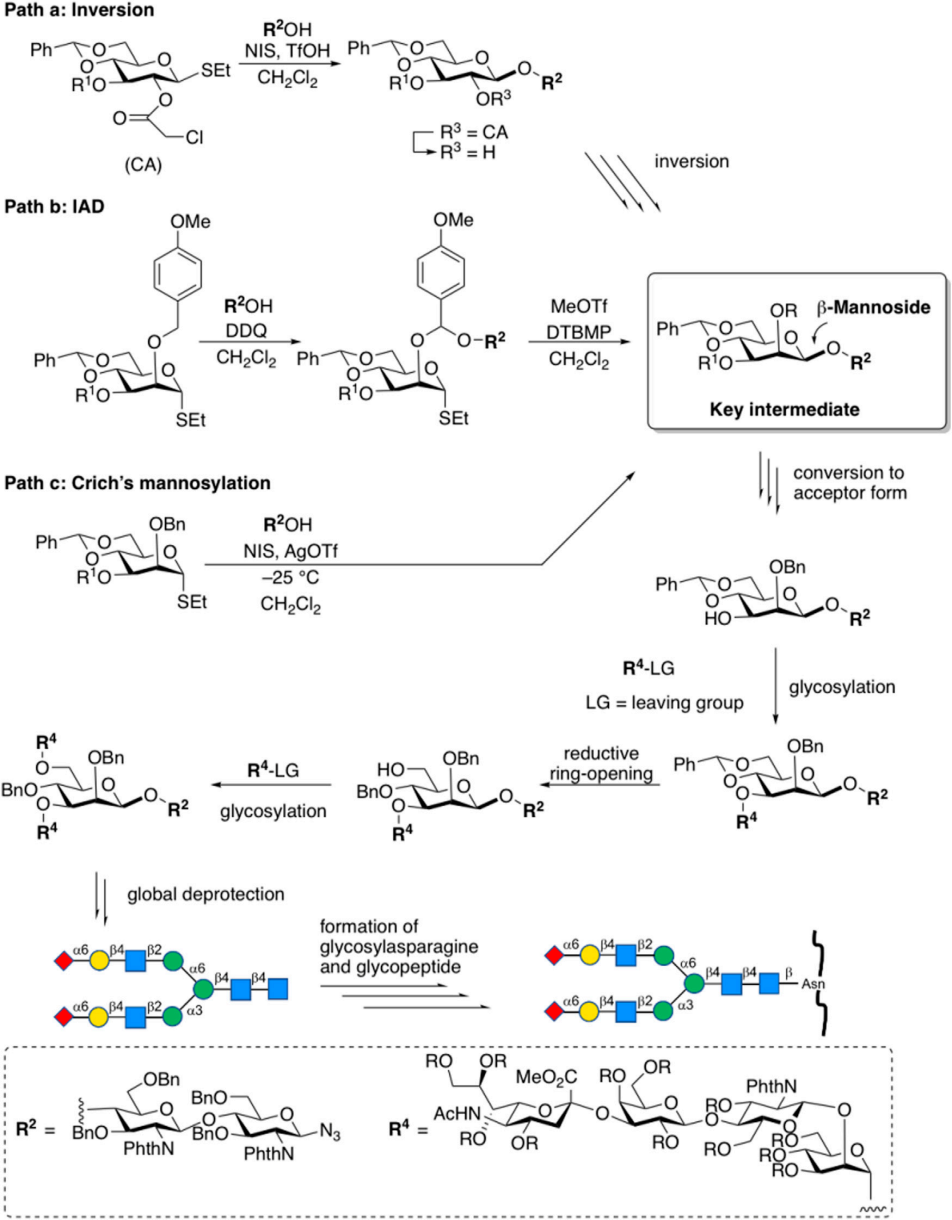
Chemical strategies for the construction of β-d-mannosidic bonds in complex-type *N*-glycans.

The stereoselective construction of β-D-mannosidic bonds has always been a hot topic as one of the most difficult issues in glycochemistry (Tsutsui et al., 2020; Ding et al., 2018; Zhong et al., 2021). Stereo electronic effects such as the anomeric effect and C (2)-OH axial substitution of mannose is both beneficial to the formation of α-configuration products. For the construction of the core structure of *N*-glycan, a variety of effective methods has been reported. To sum up, there are three main chemical strategies so far for the construction of β-D-mannosidic bonds to synthesize complex-type *N*-glycans, including the β-glycosylation-inversion strategy (Matsuo et al., 1999), intramolecular aglycon delivery (IAD) (Barresi and Hindsgaul, 1991), and 4,6-*O*-benzylidene protecting strategy (Crich’s mannosylation) (Crich and Sun, 1996) (Figure 4). Because of the efficiency of the simple intermolecular reaction, and the predominant β-selectivity of Crich’s mannosylation (for a recent example of β-glycosylation-double inversion strategy, see Ishii et al., 2018) (for an example of an improved IAD, see Ishiwata et al., 2008), 4,6-*O*-benzylidene-protected glycosyl donors were trends to be selected, although the other methods also afford β-D-mannose in the acceptable stereoselectivity. Furthermore, some efforts have also been made to optimize this strategy (For example, the gold(I)-catalyzed glycosylation reaction with glycosyl ortho-alkynyl benzoates as donors, see: Zhu and Yu, 2015).

The non-reducing terminal modification of *N*-glycans with sialic acids has a great impact on the physiological and pathological function of glycoproteins. Sialic acids on proteins of mammalian cell surface can be divided into *N*-acetylneuraminic acid (Neu5Ac) and *N*-glycolylneuraminic acid (Neu5Gc), which link to 3 or 6 positions of D-galactopyranose residue of *N*-glycan through α-glycosidic linkages (Shirakawa et al., 2021a). The stereoselective formation of sialo side possessing a 3-deoxy-keto-pyranoside structure is extremely difficult since natural sialo side in *N*-glycan is in equatorial glycoside form, which can’t be controlled by both anomeric effect and neighboring group participation when missing stereo-directing hydroxy group at 3-position of sialic acid. Scientists have applied chemical sialylation by effective stereoselective methodology including the effect of solvent (Kanie et al., 1988), the introduction of auxiliary at C3-position, functionality at C5-*N* and C1-*O* positions (for a recent review, see Vibhute et al., 2021), C4-*O*-,C5-*N*-oxazoline (Tanaka et al., 2020) and C1-*O*-,5-*N*-macrocyclic constrain (Komura et al., 2019) to the synthesis of the non-reducing terminal structure of *N*-glycan as well as cell surface glycolipid and polysialic acid structures. Ando’s group designed bicyclic sialyl donors tethered by macrocycle formation between C1 and C5 to realize selectively α-sialylation in 2019, through constrained oxocarbenium ions, which could be applied to a wide range of substrates from simple aglycone to glycosyl acceptors, giving high yields and complete stereo control (Komura et al., 2019). In the recent example for *N*-glycan structure, 1-*O*-picolinyl-5-azido thiosialosides have been used as α-selective glycosyl donors followed by using a “latent-active” protocol such as MPEP (*o*-(*p*-methoxyphenyl ethynyl)phenyl) glycosylation strategies for further elongation of reducing side residues (Chen et al., 2019).

**FIGURE 5:**
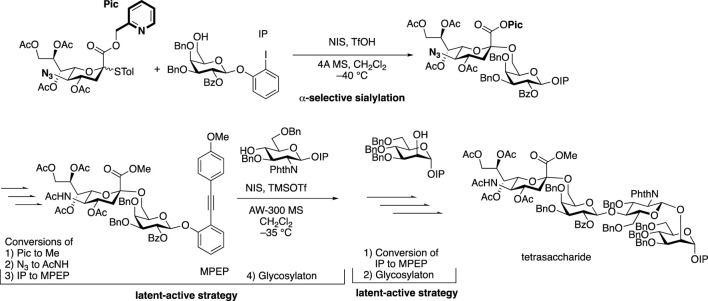
α-Sialylation for α-2,6-linkage to galactose residue followed by latent-active strategies toward the synthesis of tetrasaccharide branch (type-I) in complex-type *N*-glycan.

The assembly strategy of glycans, in other words, by using existing glycosylation methods to construct complex-type glycan structures is another important part of glycochemistry. In 2009, Danishefsky et al. adopted a linear strategy to synthesize the I-type antenna sialylated *N*-glycans and coupled core heptasaccharide with mannose and fucose units (Wang et al., 2009), and Sun et al. recently reported the linear strategy in fewer steps with higher selectivity (Chen et al., 2019) (Figure 5). On the other hand, Wang et al. developed a three-component one-pot synthetic strategy to assembly the core hyperbranched hexasaccharide of *N*-glycans linked to chloroviruses ATCV-1 using thioglycosides as building blocks, specifically attaching two xylose donors and a rhamnose donor to a trisaccharide, with regio- and stereo-selectivity (Wang Y. S. et al., 2019). Most recently, a convergent strategy was applied to synthesize the representative glycoform in homogeneous human E-cadherin *N*-linked glycopeptides containing a core tri- or tetra-saccharide and biantennary moiety, which attached the antennas to *N*-glycan by coupling glycosyl fluoride or thioglycoside donors with core oligosaccharide, and showed stereoselectivity controls (Zeng et al., 2020).

**FIGURE 6:**
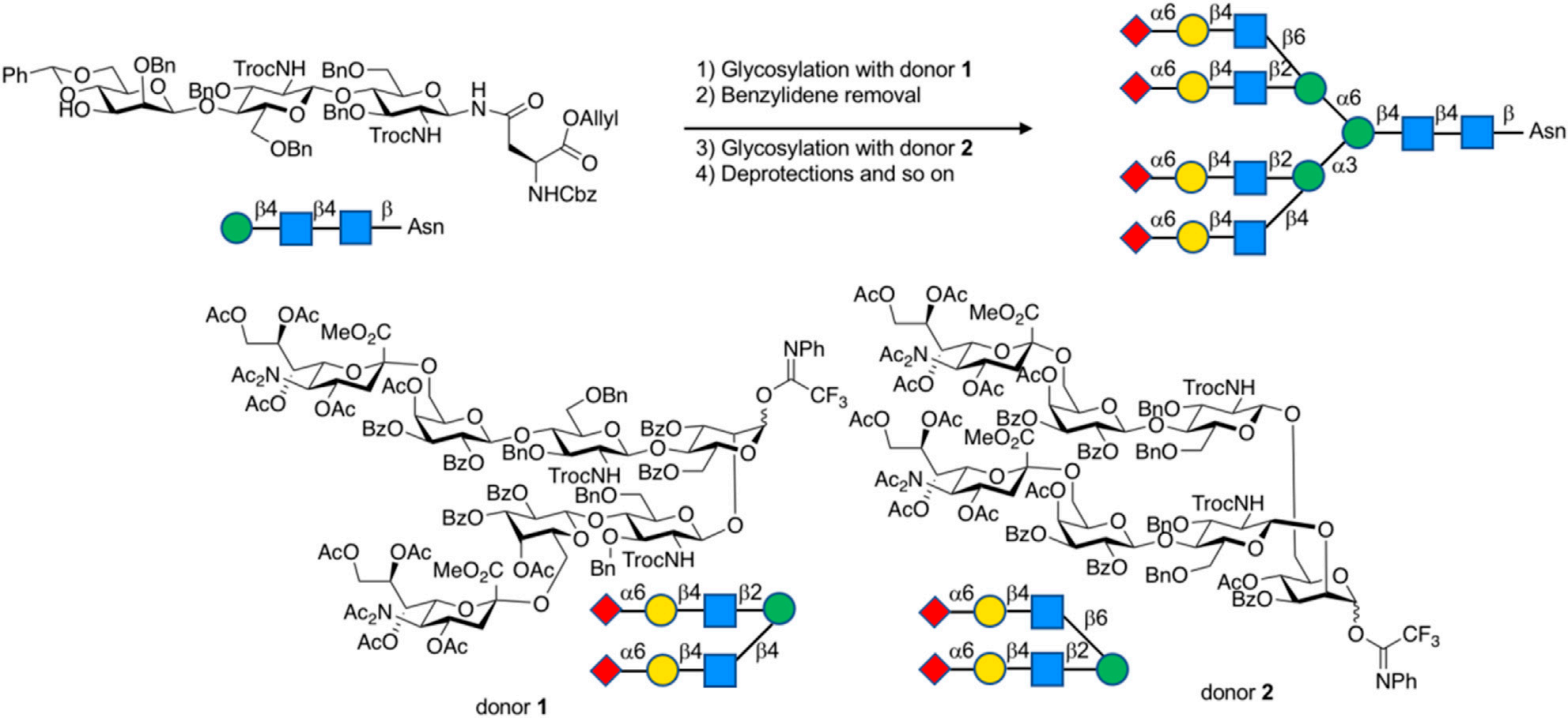
Microfluidic assembly of complex-type *N*-glycans.

In 2019, the microfluidic systems combined with convergent synthetic routes had been applied to produce complex-type *N*-glycans (Manabe, 2019). In this strategy, glycosylated fragments are reproducibly obtained under strict control of reaction conditions by using microflow systems, followed by the assembly of fragments into desired oligosaccharide backbone structures through convergent synthetic routes. Overall, the synthesis of *N*-glycans could be accomplished in relatively short steps. The key to this strategy is how to achieve a satisfactory level of efficiency in glycosylation among less reactive large fragments. It shows that amide groups (NHAc) form intermolecular hydrogen bonds to reduce the reactivity. The glycosylation reactivity could be markedly improved by protecting them as imides (NAc_2_) (Demchenko and Boons, 1998). A relatively high yield of the desired product can be achieved by using ether solvent for coordination stabilization of the oxocarbenium ion intermediate even in poorly reactive glycosylation. In addition, this strategy has successfully improved the stereoselectivity by carefully altering protection strategies. The strategy was applied to the synthesis of H antigen trisaccharide (Manabe, 2020) as well as 3- and 6-α-sialylated tetraantennary *N*-glycans for H1N1 neuraminidase recognition (Manabe, 2021) very recently (Figure 6).

**FIGURE 7:**
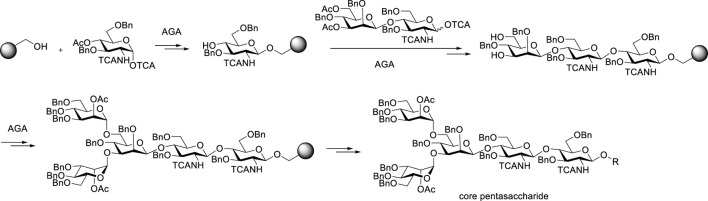
Automated glycan assembly of core pentasaccharide present in complex-type *N*-glycans.

The automated solid-phase strategy first reported in 2001 (Sabbavarapu Seeberger et al., 2001) has been applied to the *N*-glycan core structures (Le Mai Hoanget al., 2019) (Figure 7). As with the automated synthesis of peptides or nucleic acids, glycosyl acceptors are linked to resins via a connecting arm, which is called a linker. Each desired linkage is completed by adding a glycosyl donor, and then the temporary protecting group has to be removed. The procedure is repeated and the solid support serves to keep the growing chain in a form that can be removed from the reaction mixture by filtration. The linker is disconnected upon completion of the synthesis. Finally, the fully assembled product is cut from the resin after purifying by simple filtration. Since donors in each step have overdosed, acceptors supported on resins could be completely glycosylated as far as possible, which leads to high overall efficiency in theory. Using this automated glycan assembly (AGA) strategy, the automatic synthesis has been applied to the chemical synthesis of core pentasaccharide of *N*-glycans, arabinomannan polysaccharides (Pardo-Vargas et al., 2019), chitooligosaccharides (Tyrikos-Ergas et al., 2021), galactofuranoses (Sabbavarapu and Seeberger, 2021) and starch and glycogen polysaccharides (Zhu et al., 2021).

### Chemoenzymatic Strategies

Chemists have begun exploring the combination of biocatalytic and modern chemical methods to provide complex carbohydrates in a more efficient way since the 1990s. For example, Unverzagt reported the synthesis of a sialylated biantennary *N*-glycan in 2002 (Unverzagtet al., 2002). Such a chemoenzymatic approach not only shows the flexibility of chemical methods but also the high stereoselectivity and regioselectivity of enzymatic reactions. In general, there are two types of chemoenzymatic methods based on the sequence of reaction events. One starts from the chemical synthesis to generate substrate analogs for enzymatic extension, and the other is to apply enzymatic synthesis first before chemical diversification takes place. In the early stage, many studies focused on the chemoenzymatic synthesis of simple symmetrical *N*-glycans (recent review, see Chao et al., 2020; Li and Wang, 2018).

### Top-Down Strategy From Synthetic Large Glycan (One Large Precursor) Using Glycosidases

**FIGURE 8:**
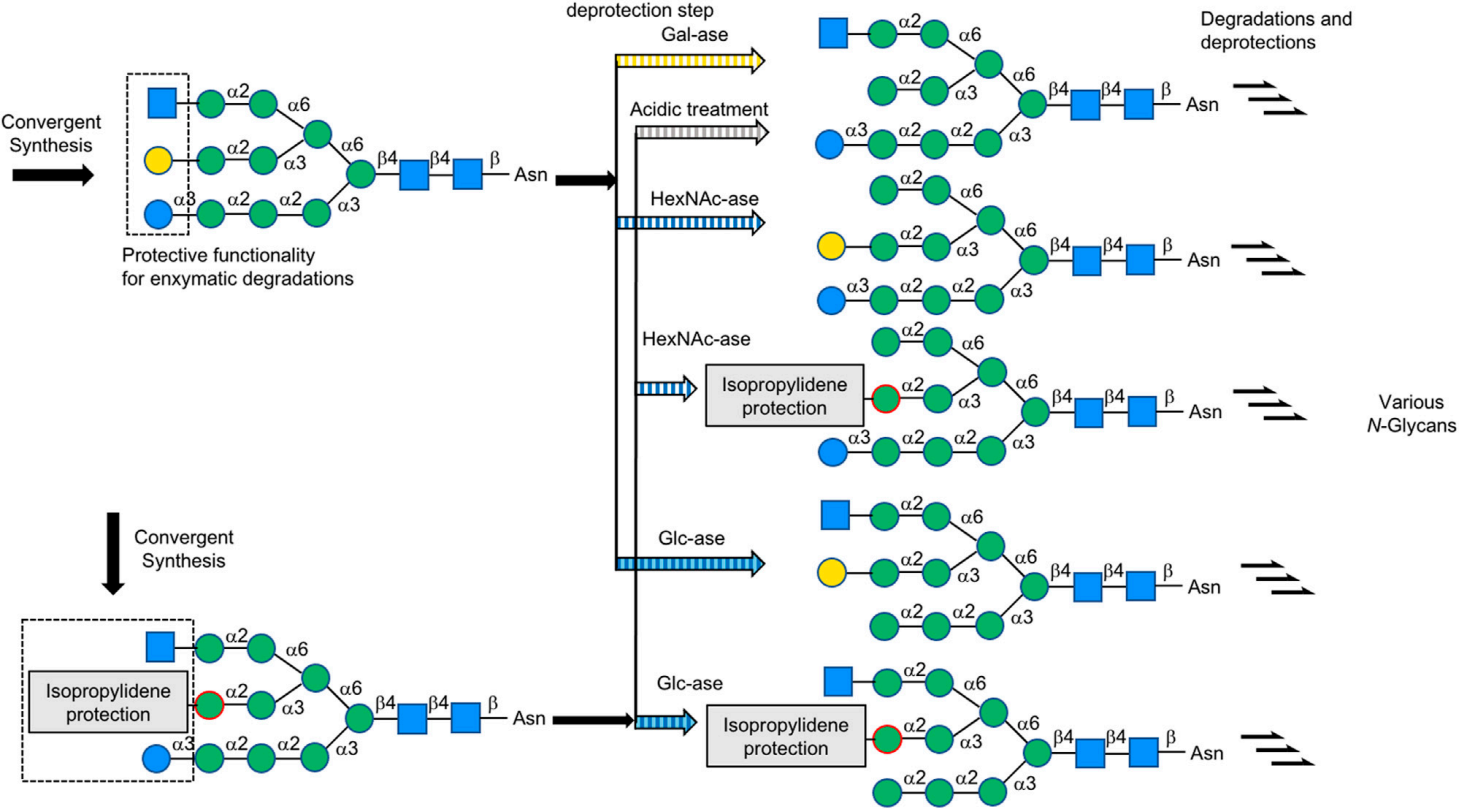
Top to down strategy for complex-type *N*-glycans.

In 2013, Ito’s group first reported a “top-down” chemoenzymatic strategy to construct a high-mannose type *N*-glycan library from a designed precursor, a tetradecasaccharide modified with Glc, Gal, and GlcNAc terminal sugar residues, followed by trimming this precursor into a variety of high-mannose type glycans by glycosidases. For instance, D-glucose moiety was removed by glucosidase Ⅱ and D-galactose by β-galactosidase, while *N*-acetyl-D-glucosamine was removed by β-HexNAc’ase (Koizumi et al., 2013) (Figure 8). They then developed the second generation precursor in 2016, substituted acetal protecting group directly on Mannose residue instead of Gal protection in that branch, due to the difficulty of galactosidase digestion (Fujikawa et al., 2016). So far, this strategy has been employed in the synthesis of high-mannose type *N*-glycans. Wang et al. also applied a top-down strategy starting from Soybean flour Man_9_GlcNAc_2_Asn and Hen egg yolk sialylglycopeptide (SGP) to high-mannose and complex-type *N*-glycans (Wang Y. S. et al., 2019).

### Elongation and Branching Strategy From Synthetic Small Glycan Using Glycosyltransferases

**FIGURE 9:**
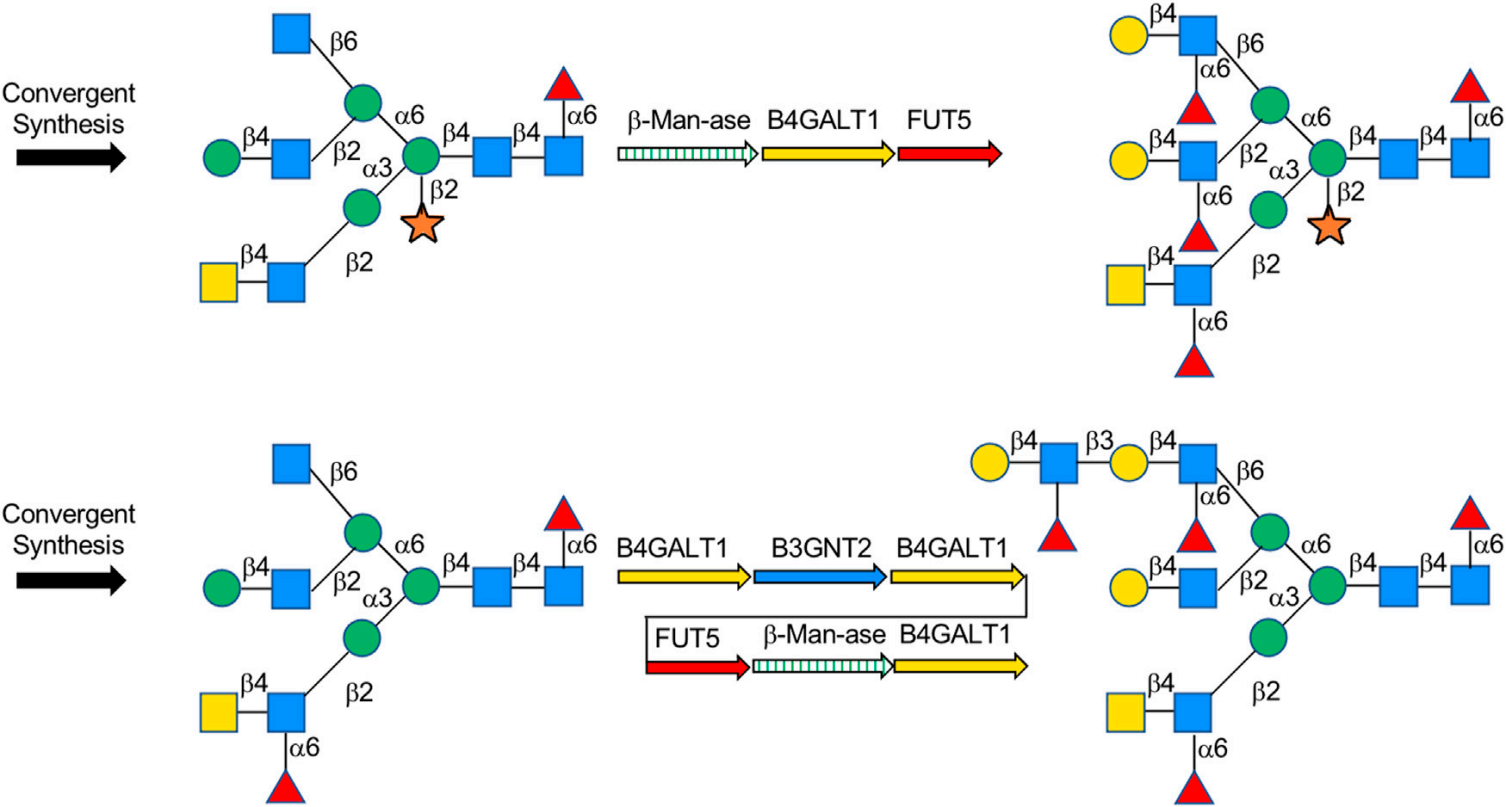
Chemoenzymatic synthesis of complex-type *N*-glycans.

A lot of explorations have been done by Boons on the chemoenzymatic synthesis of complex-type *N*-glycans. In his previous explorations, Boons developed a common glycan precursor first by using modern chemical methods, followed by the enzymatic extension of elongation and branching of *N*-glycans. For example, in 2013, a general strategy for chemoenzymatic synthesis of asymmetrically branched *N*-glycans was reported, and 14 tri-antennary *N*-glycans were obtained. In this strategy, the precursor decasaccharide was prepared from the core pentasaccharide that at potential branching positions was modified by orthogonal protecting groups and then modified branch-specifically by glycosyltransferases (Wang et al., 2013). Recently, Boons’ group synthesized *N*-glycan heptasaccharide precursors of the Parasite *Schistosoma mansoni* by chemical glycosylation method, achieved xylose modification by β-1,2-xylosyltransferase XYLT, and transferred to multi-antennary glycans enzymatically via β-Man-ase, B4GalT1 and FUT5 (Srivastava et al., 2021) (Figure 9). Weiss et al. also applied the synthesis of the rare biantennary *N*-glycans with Gal-β1,4-linked bisecting GlcNAc motif found in IgG by using a chemical modular approach to assemble the core bisected *N*-glycans followed by simple enzymatic antenna modifications with β1,4-galactosyltransferase (Weiss et al., 2020). A previous application of this strategy was also shown in order to obtain various *N*-glycan structures including Neu5Ac/Gc and core-fucoses. It features the initial convergent chemical assembly of core oligosaccharide precursors and following enzymatic extension catalyzed by an alternative combination of four robust glycosyltransferases, PmST1m, Hpα1,3FT, B4GALT1, and Pd2,6ST, to yield a library of complex-type *N*-glycans. (Li et al., 2015).

**FIGURE 10:**
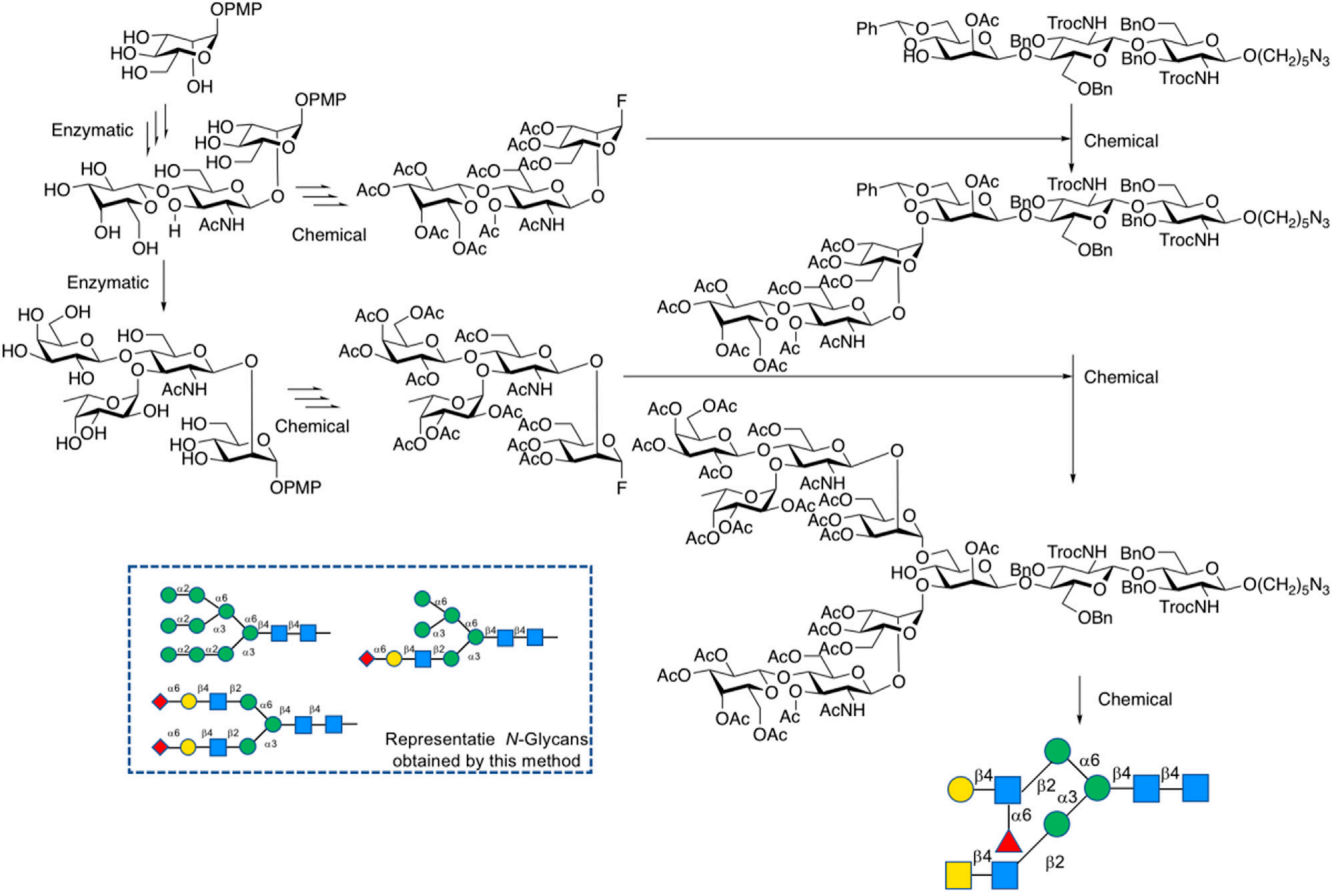
Chemo-enzymatic synthesis of modules.

Shivatare et al. prepared *N*-glycans of hybrid, high-mannose, and complex types for microarray and binding analysis of HIV antibodies first by chemical glycosylation to obtain building blocks, followed by a modular chemoenzymatic strategy to add antennas using various glycosyltransferases including β-1,4-galactosyltransferases, α-2,3/2,6-sialyltransferases and α-1,3/1,2-fucosyltransferases (Shivatare et al., 2016) (Figure 10).

**FIGURE 11:**
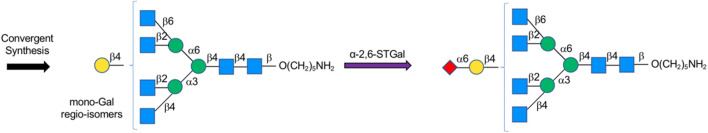
Asymmetric glycan common-substrate (AGCS).

Pawar et al. constructed *N*-glycan libraries by synthesizing oligosaccharide building blocks chemically in Asymmetric Glycan as Common-substrate Strategy (AGCS), followed by introducing fucose and sialic motifs to core structures via glycosyltransferases FUT, α-2,3-STGal, or α-2,6-STGal (Pawar et al., 2020) (Figure 11).

**FIGURE 12:**
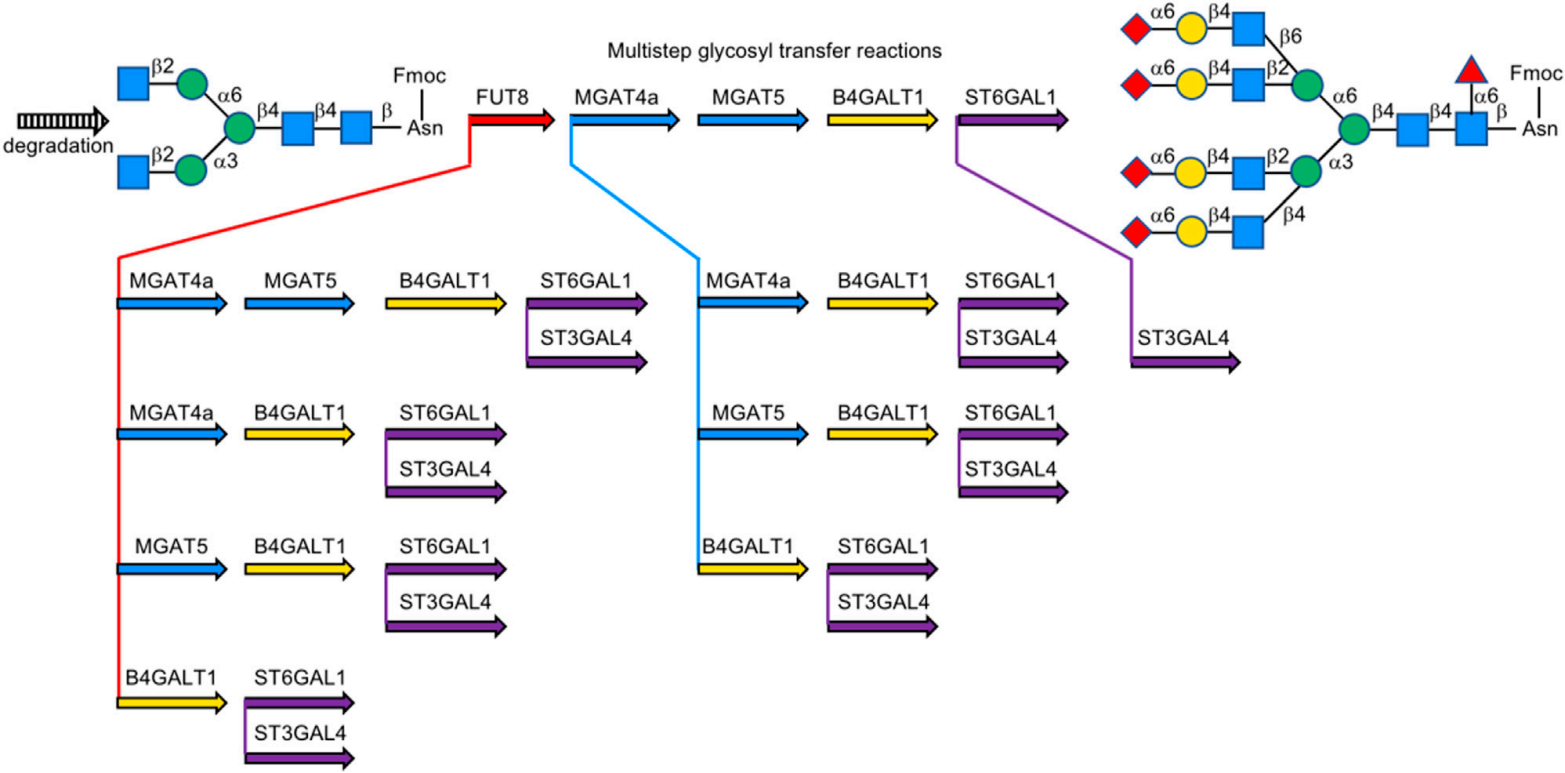
Generation of complex-type naturally occurring *N*-glycans library.

Cummings et al. enzymatically generated a multi-antennary oligosaccharide library containing 32 complex-type *N*-glycans from a natural source, in which recombinant glycosyltransferases (B4GALT1, ST3GAL4, ST6GAL1, MGAT, and FUT8) were used to expand synthesis from a precursor. Desired sialylated, fucosylated, and mannosylated terminal modifications and branching were realized on a biantennary GlcNAc-terminated structure (Gao et al., 2019) (Figure 12).

### Strategy Using Endo-Type Enzymes and Mutants

**FIGURE 13:**
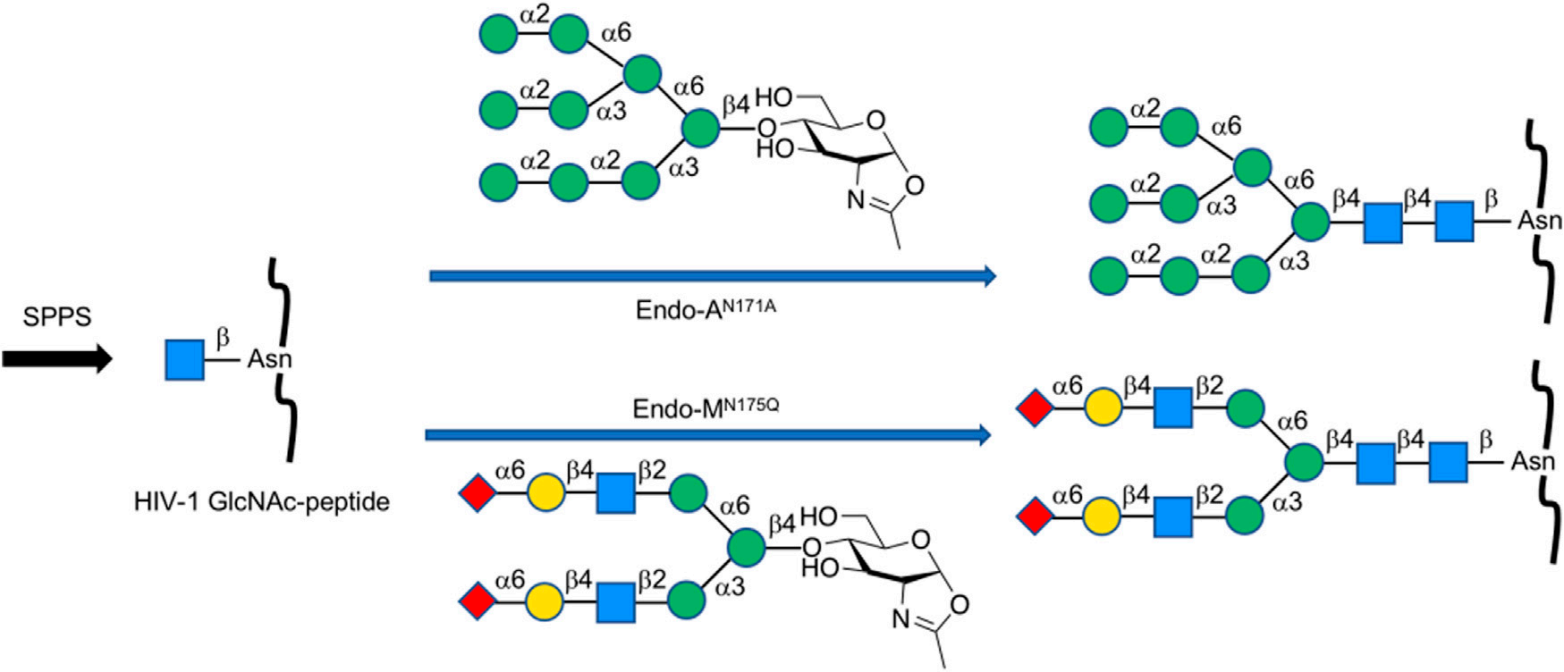
Chemoenzymatic synthesis of homogeneous *N*-glycan-linked HIV-1 glycopeptides.

**FIGURE 14:**
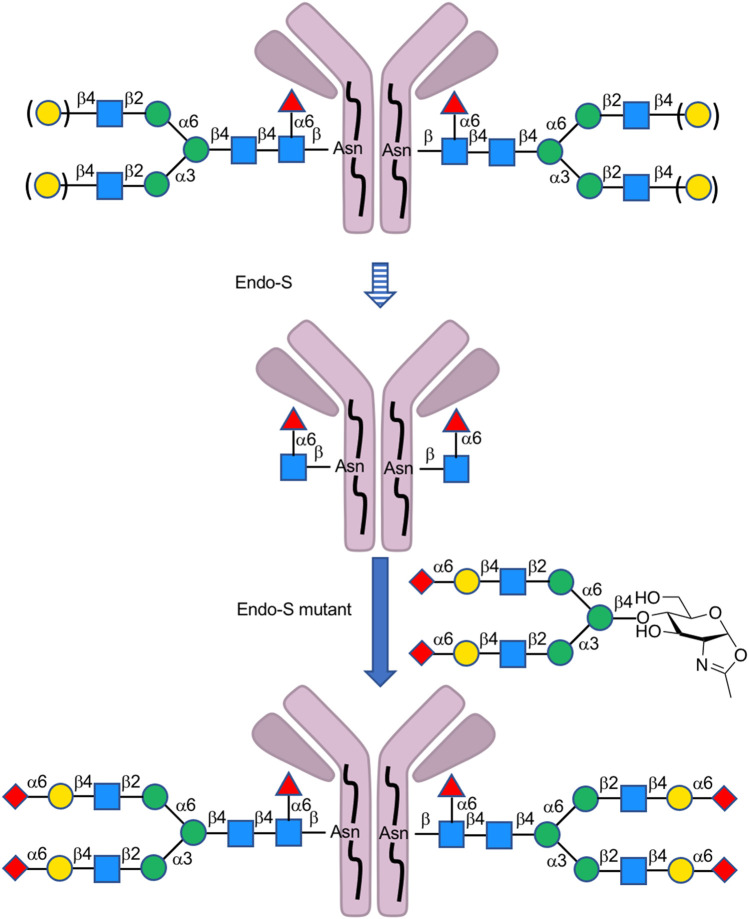
Preparation of homogeneous *N*-glycan-linked antibody-mediated by Endo-type enzymes.

**FIGURE 15:**
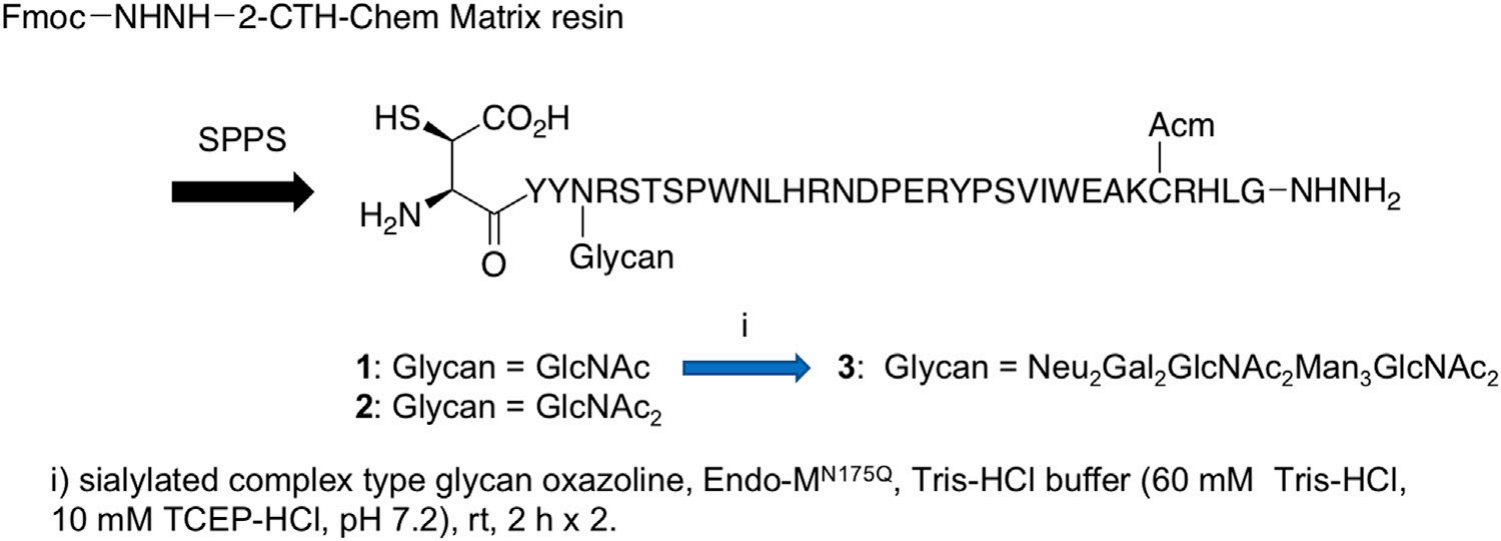
Preparation of IL-17A glycopeptides.

Endo-β-*N*-acetylglucosaminidases, known as endo-type enzymes of wild types and mutants, are often used in the synthesis of *N*-linked glycopeptides, which perform activities of deglycosylation by breaking between GlcNAcs in *N*-linked chitobiosemoiety, and transglycosylation to GlcNAc-linked peptides. These enzymes are found in a wide range of species, such as Endo-A from *Arthrobacter*, Endo-M from *Mucor hiemalis,* and Endo-S from *Streptococcus pyogenes*. Since endo-α-*N*-acetylglucosaminidases are only specific to *O*-glycans, endo-β-*N*-acetylglucosaminidases (ENGases) are considered the main endo-type to prepare homogeneous *N*-linked glycoprotein. For the transfer of oligosaccharides back to the cleaved structure, the mutants mostly use glycan oxazolines as donor substrates and have stereoselective control on substrates. Recently, Wang et al. applied this procedure to synthesize HIV-1 glycopeptides through the preparation of GlcNAc-peptide precursor by using solid-phase peptide synthesis (SPPS) and followed by the connection of *N*-glycan to the precursor using the *N*-glycan oxazoline donor via endo-glycosidase (Endo-A or Endo-M) mutant catalysis (Zong et al., 2020) (Figure 13). They also reported recombinant Endo-S and mutants that proceeded deglycosylation to convert structurally heterogeneous sugar chains to GlcNAc-IgG acceptor and remodeling to homogeneously *N*-glycosylated Fc fragments on rituximab (Wang L.-X. et al., 2019) (Figure 14). Dong et al. introduced *N*-linked oligosaccharides including GlcNAc or GlcNAc_2_ by chemical glycosylation with asparagine residue on peptide precursor as well as complex-type sialyl *N*-glycan by transfer using Endo-M mutant onto IL-17A peptide (Li H. et al., 2021) (Figure 15). It is notable as another previous example of this strategy that the chemo-enzymatic synthesis of *N*-glycopeptide structural motif of haptoglobin glycopeptides had been achieved by a total chemical synthesis of the highly branched oligosaccharide oxazoline donors through fragment couplings and an alternative wild-type Endo A-catalyzed transglycosylation of the oxazolines in the presence of bisecting GlcNAc to GlcNAc-introduced haptoglobin glycopeptide fragment (Yang et al., 2018). Interestingly, wild-type Endo A could be also applicable to the preparation of an *N*-linked glycoprotein that contains M6P-terminated glycans catalysis for the treatment of lysosomal storage disorders (LSDs) (Priyanka, et al., 2016).

### OST Strategy

**FIGURE 16:**
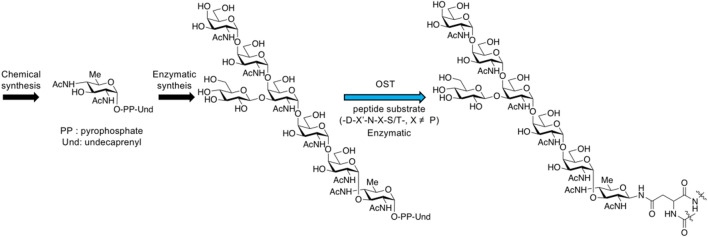
*N*-glycan synthesis through bacterial biosynthesis including oligosaccharyltransferase reaction.

Oligosaccharyltransferases (OST) usually exist in prokaryotes (bacteria and archaea) and eukaryotes in the form of complex, playing a key role in *N*-glycosylation, with the catalysis of transferring oligosaccharide chains onto protein substrates. Single subunit OST was found in the bacterial pathway of glycoprotein biosynthesis, such as PglB from *Campylobacter jejuni* (Wacker et al., 2002; Deshpande et al., 2008; Lizak et al., 2011; Jinnelov et al., 2017; Poljak et al., 2017; Mohanty et al., 2020; Schjoldager et al., 2020) and TbSTT3A from *Trypanosoma brucei* for *N*-glycan (Wilson et al., 2021). The archaeal pathway also includes the single subunit OST such as AglB (Meyer and Albers, 2014; recent review, see Eichler, 2020), whose X-ray structure from *Archaeoglobusfulgidus* has been reported very recently (Taguchi et al., 2021). The most studied PglB can transfer the highly conserved heptasaccharide composed of GalNAc_5_Glc_1_Bac_1_ (Bac: bacillosamine, 2,4-acetamide-2.4.6-trideoxy-D-glucose) to proteins in the periplasm of *C. jejuni*. Chemical and enzymatic synthesis of *N*-glycans and glycopeptide synthesis using OST have been already reported (Glover et al., 2005; Amin et al., 2007; Lee et al., 2009; Lukose et al., 2015; Ishiwata et al., 2015) (Figure 16).

In humans, mutations of gene encoding OST complex may cause some diseases, like congenital disorders of glycosylation (CDGs) (Ganetzky et al., 2017), making OST an important enzyme for human glycosyl transfer to maintain normal physiological functions, but there are no examples for using mammalian OST complex to produce the good quantity of *N*-glycoprotein effectively (Kay et al., 2019).

### Enzymatic and Chemoenzymatic Strategy From Natural Resources (such as SGP) Using Glycosidases With/Without Glycosyltransferases

Chemical synthesis of precursors for enzymatic modification is still not efficient enough because it often requires many steps including cumbersome protection/deprotection, resulting in product loss, and hindering the preparation of various types of asymmetric *N*-glycans. Researchers have discovered starting materials from natural sources, such as sialylglycopeptide (SGP), and have greatly optimized the isolation method for gram quantities of homogeneous SGP from commercially available egg yolk powder. Hamilton et al. generated a library containing complex-type asymmetric and multi-antennary *N*-glycans for microarray analysis, by deglycosylation of biosynthetic precursor glycoproteins and lipid-linked oligosaccharides from natural sources (yeast- and bacteria-derived precursors) and structurally remodeling by GnTⅠ, GnTⅡ, and GnTⅣ as well as the following early mammalian glycosylation pathway (Hamilton et al., 2017). Wang et al. reported chemoenzymatic strategy on natural *N*-glycans from soybean flour and SGP from egg yolks and trimmed enzymatically by α-mannosidases to generate high-mannose glycan library also for microarray analysis (Toonstra et al., 2018).

**FIGURE 17:**
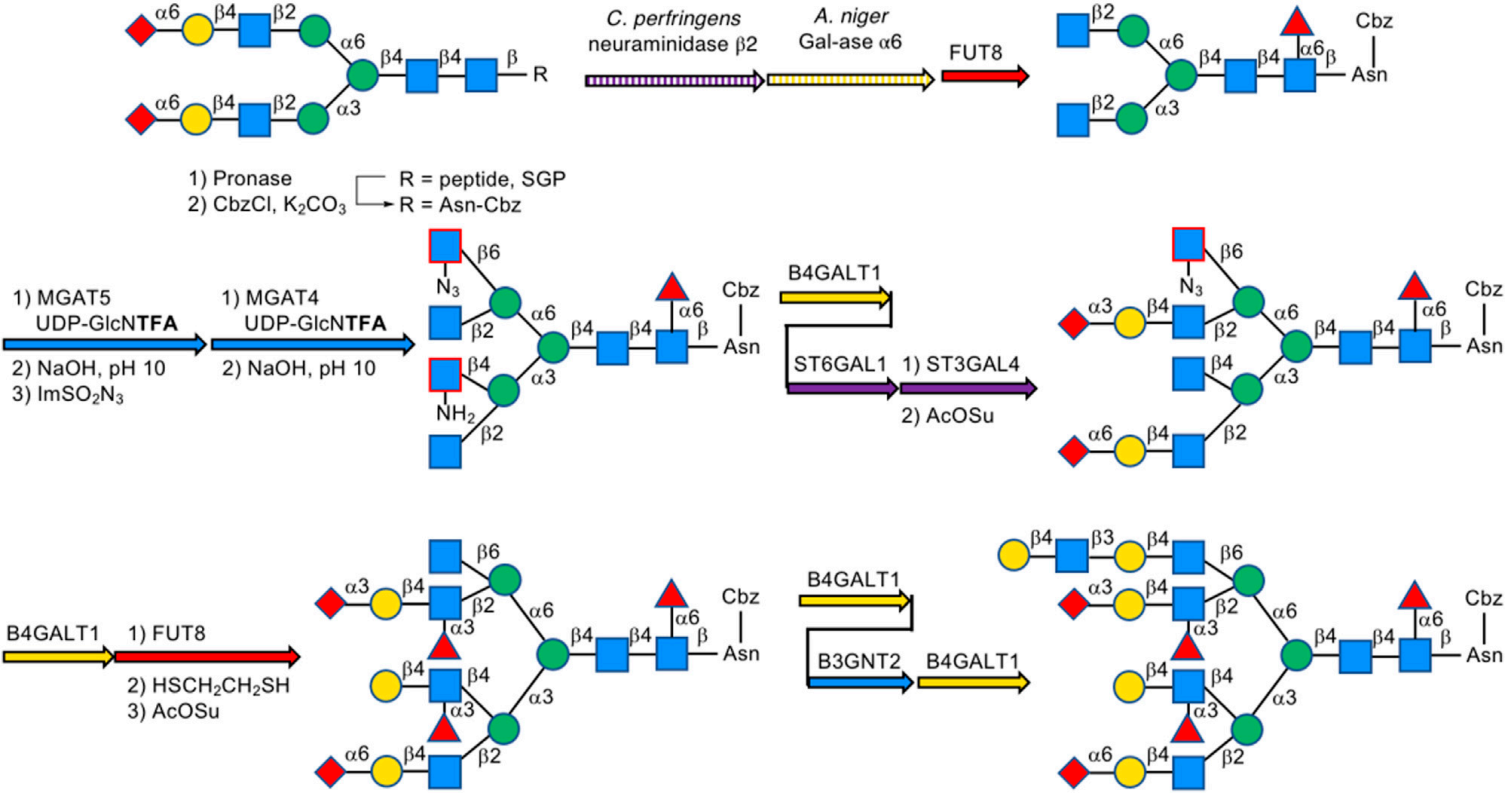
Stop and Go strategy for multi-antennary *N*-glycans; For MGAT4/5&B3GNT2, B4GALT1, FUT8, and ST3/6GAL1/4, UDP-GlcNAc, UDP-Gal, GDP-Fuc, and CMP-Neu5Ac were used as the donor, respectively, unless otherwise noted.

In 2019, Boons et al. described a biomimetic strategy called Stop and Go strategy, in which SGP can be converted through degradations and glycosyl transfer reactions including the introduction of nonreactive chemically modified residue and its chemical modifications to activate form from unreactive one and further enzymatic steps into multi-antennary *N*-glycans that at each arm can be uniquely extended by glycosyltransferases to obtain highly complex asymmetrically branched *N*-glycans. By using this strategy, Boons has successfully accomplished the chemoenzymatic synthesis of several complex-type *N*-glycans found in human pathological tissues, like bi-antennary *N*-glycans observed on ovarian cancer cell lines as well as tetra-antennary *N*-glycans observed on human cytolytic T lymphocytes (Liu et al., 2019) (Figure 17).

In 2021, Wang et al. prepared the complex-type asialo-nonasaccharide of SARS-Cov-2 Spike Receptor-Binding Domain (RBD) using Boon’s reported enzymatically trimming approach on SGP in 2017, after enzymolysis of neuraminidase and pronase to remove Neu5Ac and peptide, followed by transferring into glycosyl amine under Kochetkov amination condition and coupling with an acid group of RBD peptide fragment (Ye et al., 2021).

## Concluding Remarks

Most of the human cell surface and secreted proteins are modified by complex-type *N*-glycans, which not only affect the structure and function of proteins but also participate in the signal transduction of tumor cells. In the field of biomedical applications, complex-type *N*-glycans and their glycoconjugates show broad prospects, such as anti-tumor kits and vaccines. However, unlike other important biopolymers such as DNA/RNA and proteins, the biosynthesis of glycans is neither driven by templates nor genetically encoded. This biosynthetic nature of *N*-glycan as a secondary metabolite leads directly to the micro-heterogeneity of the naturally obtained glycans, which means that it is difficult to obtain enough homogeneous complex-type *N*-glycans by means of separation and isolation. In order to learn more about the properties of *N*-linked glycopeptides and use them in the production of peptide vaccines, it is necessary to develop a universal and efficient method to prepare homogeneous *N*-glycans in addition to further optimization of purification methods from natural sources. Over the past few decades, researchers have made lots of efforts for the chemical and chemoenzymatic strategies for well-defined *N*-glycans. Though great progress has been made, there are still problems and difficulties to be solved urgently, which requires researchers to explore continuously in this field as the recent examples are shown here. Further improving efficiency and simplifying procedures are quite significant.
